# Quantitative serosal and mucosal optical imaging perfusion assessment in gastric conduits for esophageal surgery: an experimental study in enhanced reality

**DOI:** 10.1007/s00464-020-08077-3

**Published:** 2020-10-07

**Authors:** Manuel Barberio, Eric Felli, Margherita Pizzicannella, Vincent Agnus, Mahdi Al-Taher, Emilie Seyller, Yusef Moulla, Boris Jansen-Winkeln, Ines Gockel, Jacques Marescaux, Michele Diana

**Affiliations:** 1 Institute of Image-Guided Surgery, IHU-Strasbourg, 1 place de l’Hôpital, 67091 Strasbourg, France; 2 Department of Visceral, Transplant, Thoracic, and Vascular Surgery, University Hospital of Leipzig, Leipzig, Germany; 3 Research Institute Against Digestive Cancer (IRCAD), Strasbourg, France; 4 ICube Lab, Photonics Instrumentation for Health, Strasbourg, France; 5 Department of General, Digestive and Endocrine Surgery, University Hospital of Strasbourg, Strasbourg, France

**Keywords:** Esophagectomy, Esophageal resection, Gastric conduit, Blood flow assessment, Perfusion assessment, Hyperspectral imaging, Confocal laser endomicroscopy, Enhanced reality

## Abstract

**Introduction/objective:**

Gastric conduit (GC) is used for reconstruction after esophagectomy. Anastomotic leakage (AL) incidence remains high, given the extensive disruption of the gastric circulation. Currently, there is no reliable method to intraoperatively quantify gastric perfusion. Hyperspectral imaging (HSI) has shown its potential to quantify serosal StO_2_. Confocal laser endomicroscopy (CLE) allows for automatic mucosal microcirculation quantification as functional capillary density area (FCD-A). The aim of this study was to quantify serosal and mucosal GC’s microperfusion using HSI and CLE. Local capillary lactate (LCL) served as biomarker.

**Methods:**

GC was formed in 5 pigs and serosal StO_2_% was quantified at 3 regions of interest (ROI) using HSI: fundus (ROI-F), greater curvature (ROI-C), and pylorus (ROI-P). After intravenous injection of sodium-fluorescein (0.5 g), CLE-based mucosal microperfusion was assessed at the corresponding ROIs, and LCLs were quantified via a lactate analyzer.

**Results:**

StO_2_ and FCD-A at ROI-F (41 ± 10.6%, 3.3 ± 3.8, respectively) were significantly lower than ROI-C (68.2 ± 6.7%, *p* value: 0.005; 18.4 ± 7, *p* value: 0.01, respectively) and ROI-P (72 ± 10.4%, *p* value: 0.005; 15.7 ± 3.2 *p* value: 0.001). LCL value at ROI-F (9.6 ± 4.7 mmol/L) was significantly higher than at ROI-C (2.6 ± 1.2 mmol/L, *p* value: 0.04) and ROI-P (2.6 ± 1.3 mmol/L, *p* value: 0.04). No statistically significant difference was found in all metrics between ROI-C and ROI-P. StO_2_ correlated with FCD-A (Pearson’s *r* = 0.67). The LCL correlated negatively with both FCD-A (Spearman’s *r* =  − 0.74) and StO_2_ (Spearman’s *r* =  −  0.54).

**Conclusions:**

GC formation causes a drop in serosal and mucosal fundic perfusion. HSI and CLE correlate well and might become useful intraoperative tools.

Esophagectomy is a major and morbid surgical procedure, which plays a central role in the multidisciplinary treatment of patients affected by esophageal neoplasia [1]. Postoperative complications negatively affect long-term oncological survival [2]. Anastomotic leakage (AL) is a particularly severe complication, which has a higher incidence following esophagectomy when compared to other digestive surgical procedures [3]. Although the etiology of anastomotic leaks (AL) is multifactorial, a well-identified risk factor is an insufficient perfusion at the anastomotic site. In fact, the resected esophagus is frequently replaced by a gastric conduit (GC), which is obtained from the tubulation of the stomach, which involves a sudden disruption of most of its blood supply, resulting in an unsatisfactory anastomotic perfusion.

The appreciation of clinical parameters, such as a serosal color or the presence of pulse, is inadequate to rule out a marginally perfused anastomosis [4]. Several technologies have been explored to intraoperatively assess gastrointestinal tract perfusion. However, the majority of them either lacks reproducibility, disrupts the surgical workflow, or provides operator-dependent results [5]. Fluorescence angiography (FA), a user-friendly technology which requires a near-infrared camera and the intravenous injection of an exogenous fluorophore, has shown promising results [6–8]. However, the absence of a validated quantification method of the fluorescence signal is one of the main drawbacks to be solved before understanding the real impact of this technique on anastomotic complications. Additionally, the need for injecting a contrast agent represents a regulatory burden, which in practice hinders the diffusion of this technique beyond research protocols and its introduction as a standard of care.

Hyperspectral imaging (HSI) is a contrast-free technology, which can provide a real-time snapshot of the chemical characteristics of a tissue. In particular, this imaging technique allows for tissue oxygen saturation (StO_2_) quantification and its utility during gastrointestinal procedures has been previously assessed [9–11]. The main shortcoming of HSI is the lack of video rate, which de facto greatly limits its usability as a surgical navigation tool. Our group previously described and validated the HYPER (HYperspectal-based Enhanced Reality) tool. HYPER results from the superimposition of the HSI-generated perfusion quantification pseudo-color map onto a real-time video of the surgical scene, thereby allowing for a precise spatial localization of the spectral information during surgical procedures. HYPER strongly correlated with robust ischemia parameters in an experimental small bowel and liver partial ischemia models [12, 13]. However, since HSI is an optical imaging modality, it exclusively allows for a superficial tissue analysis. As a result, serosal perfusion only can be quantified. It has been previously shown that the ischemia behavior within the gastrointestinal tract varies when comparing the serosal with the mucosal side [14]. Confocal laser endomicroscopy (CLE) is a high-resolution microscopic optical imaging modality, which allows for a detailed morphological assessment of the gastrointestinal mucosa. CLE potentially provides a precise snapshot of the tissue microcirculation, simultaneously giving a morphological in vivo histopathological appraisal and computing the functional capillary density area (FCD-A) index or the speed of red blood cells [15–17].

The aim of the current study was twofold: first, to demonstrate the accuracy of HYPER in a porcine gastric conduit model; second, to compare HSI serosal StO_2_ with CLE-based mucosal functional capillary density, using the ischemia surrogate, local capillary lactate (LCL), as “ground truth.”

## Methods

### Setup and experimental flow

Five adult male pigs (Large White, mean weight: 44.24 ± 7.6 kg) were involved in this study, which is part of the ELIOS protocol (Endoscopic Luminescent Imaging for Oncology Surgery), approved by both the local Ethical Committee on Animal Experimentation (ICOMETH No. 38.2016.01.085), and by the French Ministry of Superior Education and Research (MESR) (APAFIS#8721-2017013010316298-v2). Animals were managed according to French laws for animal use and care, to the directives of the European Community Council (2010/63/EU) and to ARRIVE guidelines [18].

Neither IRB approval nor written consent was needed since this is an experimental study.

The pigs were fasted preoperatively for 24 h and had free access to water. Premedication was administered 10 min before surgery, with intramuscular ketamine (20 mg/kg) and azaperone (2 mg/kg). Intravenous propofol (3 mg/kg) and rocuronium (0.8 mg/kg) were used for induction. Anesthesia was maintained with 2% isoflurane.

**Fig. 1:**
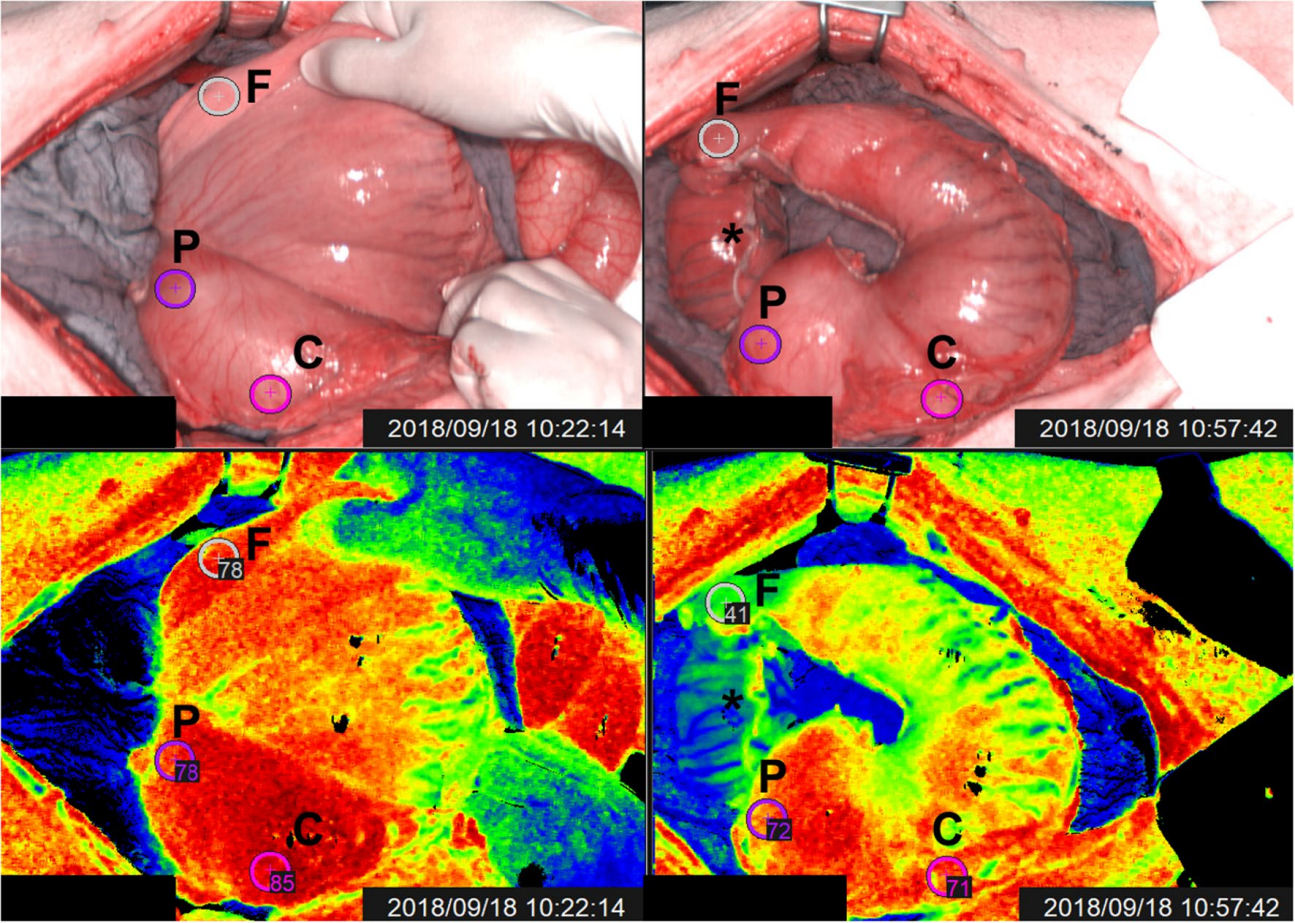
The left images show the RGB picture (top left) of the stomach prior to tubularization and its corresponding StO_2_ quantification pseudo-color image (bottom left). The images on the right depict the stomach after gastric tube creation (top right in RGB and bottom right StO_2_ quantification) with clear StO_2_ decrease exclusively at ROI-F after gastric conduit formation

Through a median laparotomy, all gastric vessels were divided using a monopolar vessel-sealing device (LigaSure Advance™, Medtronic, USA), and the right gastroepiploic artery was exclusively maintained. Successively, an approximately 4 cm wide GC was created, starting from the lesser curvature and using a surgical stapler (ENDO GIA™ equipped with 45 mm Black Reloads, Medtronic, USA) (Fig. 1).

The next step involved a hyperspectral imager (TIVITA^®^, Diaspective Vision GmbH, Germany), placed at 35–40 cm from the porcine stomach, and an HSI acquisition was performed immediately after GC formation. Using the camera-integrated software, three regions of interest (ROI) were manually selected as follows: ROI-P (pyloric region), ROI-C (greater curvature/body), and ROI-F (fundic region/future anastomosis). StO_2_ was quantified in correspondence of all ROIs (Fig. 1). As previously described [12, 13, 19], the system had been customized by adding a webcam next to the HSI objective, in order to capture a real-time video of the surgical field and superimpose static HSI pseudo-color StO_2_ quantification images onto the live video, obtaining HYPER.

**Fig. 2:**
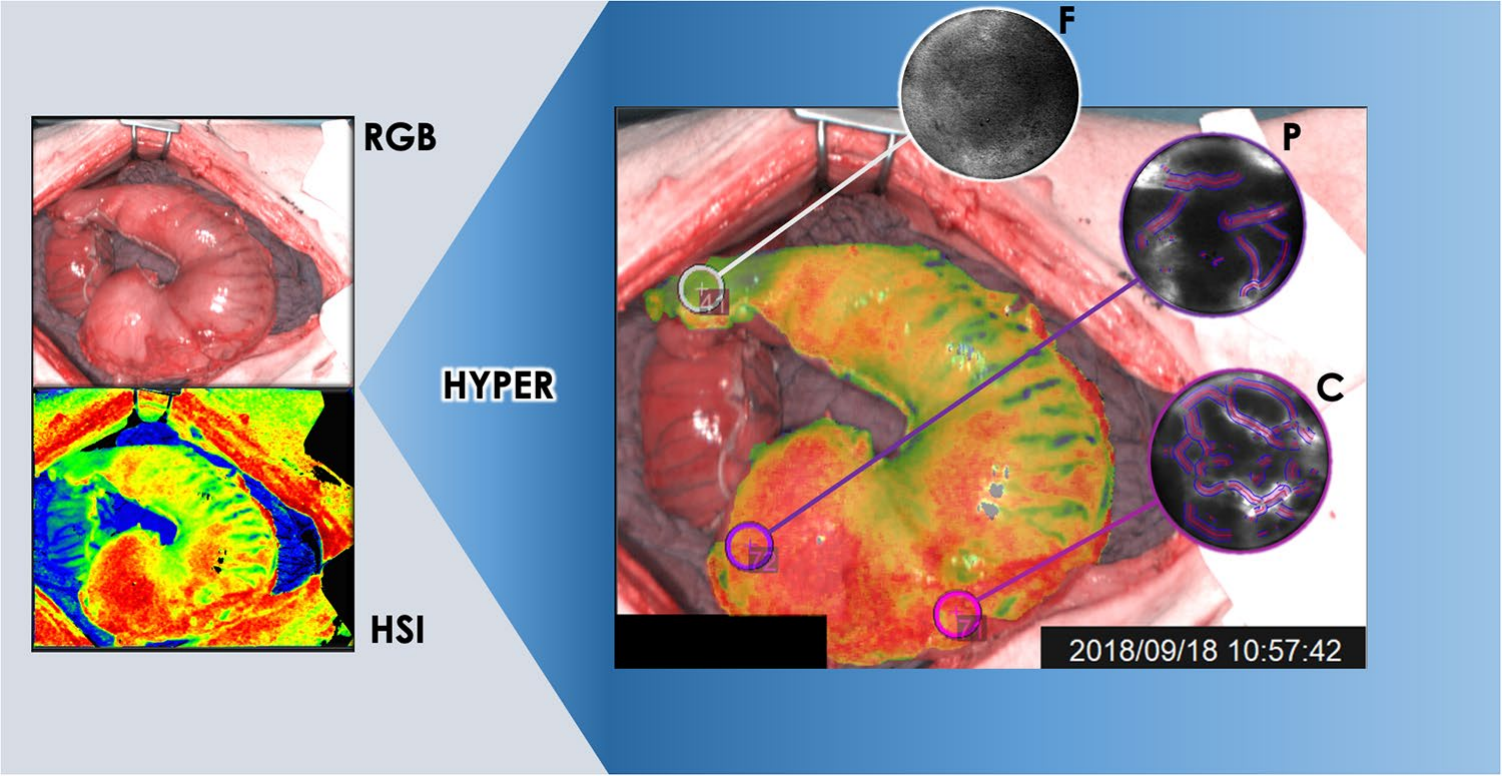
Schematic representation of HYPER, resulting from the superimposition of the StO_2_ quantification pseudo-color image on the RGB video. The ROIs are clearly visible in HYPER and this allowed for a precise mucosal scanning with CLE

Once a small opening was made in correspondence of the stapler line, 2 mL of 10% fluorescein (Fluocyne^®^, Serb, France) was administered intravenously. Subsequently, a confocal laser endomicroscopy probe (GastroFlex™ UHD, Mauna Kea Technologies, France), with a 55 to 65 μm confocal depth and a 1 μm resolution, was inserted through the stapler line’s opening and was manually driven to scan the mucosa in correspondence of the ROIs, which were displayed on the monitor using HYPER (Fig. 2). Thirty seconds long video clips were acquired. In the post-processing phase, the videos were analyzed with the IC Viewer software (version 3.8.6) (Mauna Kea Technologies, France). The software can automatically recognize elongated shapes with fluorescent contrast and classify them as vessels. Additionally, the software allows to compute the functional capillary density area (FCD-A) index. This index is calculated by multiplying the mean capillary diameter by the total vessel length, and by dividing the result by the entire area of the image.

As biological “ground truth,” local capillary lactates (LCLs) were measured [12, 16, 17, 19–22]. A full-thickness cut of the gastric wall was made in correspondence of the ROI shown by means of HYPER, and a blood drop was obtained. A portable strip-based lactate analyzer (EDGE, Apex Bio, Taiwan) was used to quantify LCL levels.

At the end of the procedure, the animals were euthanized with an intravenous injection of Pentobarbital Sodium (40 mg/kg) (Exagon^®^, AXIENCE, France), under a 5% isoflurane anesthesia.

### Prediction of local capillary lactates based on HSI StO_2_ and on the CLE FCD-A index

Similarly to previous studies on bowel perfusion assessment using HSI [12, 19], LCL prediction models based on the metrics provided by both optical imaging technologies were studied. The relationship between the biological marker (LCL) and both optical imaging output parameters (HSI StO_2_ and the CLE FCD-A index) were modeled using an exponential regression analysis. This allowed to create a prediction algorithm of LCL values from the corresponding HSI StO_2_ and the CLE-FCD-A index. The following fitting functions were found:

1. Based on HSI StO_2_:
  predicted LCL = $${\text{e}}^{\left(-0.0229 \times \text{ StO}2+ 2.93\right)}$$
2. Based on the CLE-FCD-A index:
  predicted LCL = $${\text{e}}^{(-10.5 \times \text{ FCD}-\text{A }+ 2.56)}$$

The precision of both models, indicated as the difference between the sampled LCL values and the predicted LCL values of both prediction models, was computed by the corresponding exponential equation.

### Statistical analysis

The statistical analysis was performed using the Prism 8 software (Graph Pad, USA) and the Scikit-learn Python library [23]. The parametric or non-parametric one-way ANOVA with multiple comparison tests was used as appropriate to compare the FCD-A index, LCL, and StO_2_ levels among different ROIs. Pearson’s or Spearman’s correlations were used to measure linear correlations and non-linear correlations, respectively. Exponential regression was used to investigate variables presenting a non-linear relationship. A Wilcoxon test was performed for paired comparison of lactate prediction algorithms (based on HYPER and on CLA), since data distribution was non-Gaussian. A *p* value < 0.05 was considered statistically significant.

## Results

**Fig. 3:**
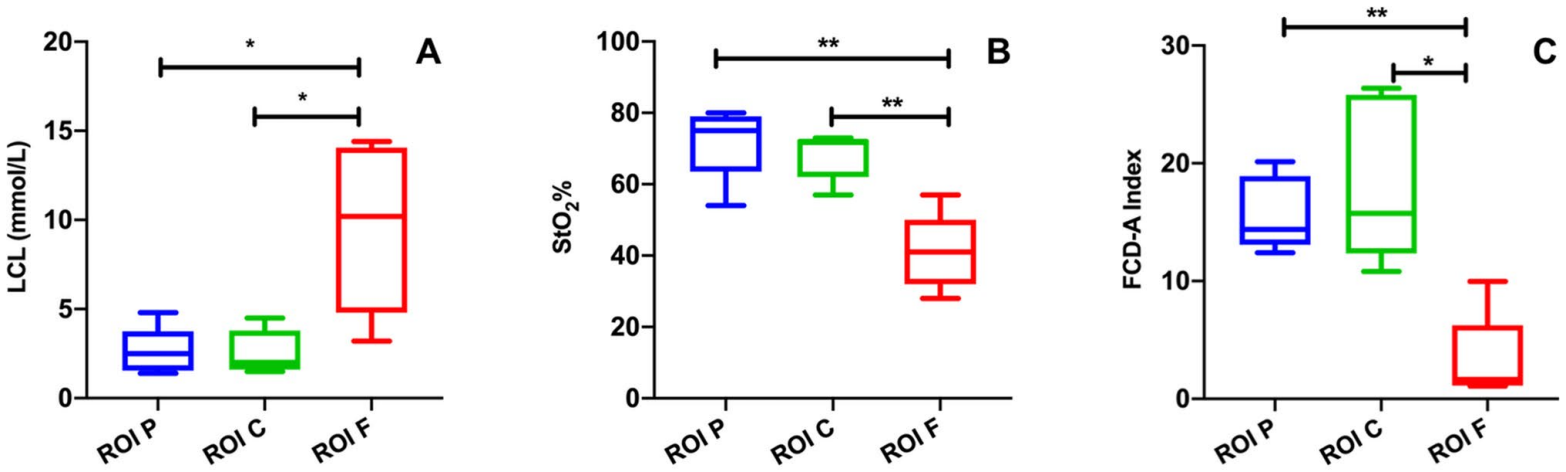
Graphical representation of the LCL and optical imaging results at the different ROIs. After gastric conduit formation, ROI-F showed a significant LCL increase (**A**) and StO_2_ (**B**), as well as a decrease in the FCD-A index (**C**) when compared to the remaining ROIs

The HYPER tool allowed for the precise identification of the ROIs onto the stomach surface, thereby guiding the CLE scanning and LCL sampling successfully. The main results are schematically represented in Fig. 3.

### HSI-based StO_2_ quantification

StO_2_ measured in correspondence of ROI-F (41 ± 10.7%) was significantly lower than at ROI-C (68.2 ± 6.7%, *p* value: 0.005) and ROI-P (72 ± 10.4%, *p* value: 0.005). There was no statistically significant difference between ROI-C and ROI-P.

### CLE-based FCD-A index

The FCD-A index at ROI-F (3.3 ± 3.8) was significantly lower when compared to ROI-C (18.4 ± 7, *p* value: 0.01) and ROI-P (15.7 ± 3.2, *p* value: 0.001). No difference was found between ROI-C and ROI-P.

### LCL quantification

The LCL value at ROI-F (9.6 ± 4.7 mmol/L) was significantly higher than at ROI-C (2.6 ± 1.2 mmol/L, *p* value: 0.04) and ROI-P (2.6 ± 1.3 mmol/L, *p* value: 0.04). ROI-C and ROI-P did not differ statistically.

### Correlations

**Fig. 4:**
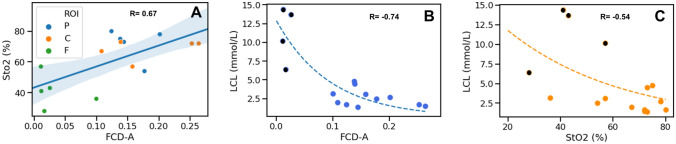
The significant positive correlation between CLE and HSI metrics is shown in (**A**). However, CLE (**B**) presents a stronger exponential relationship than StO_2_ (**C**) with LCL. In (**B**, **C**), the black spots represent the outlying ROIs, which present LCL > 6 mmol/L. Those ROIs consistently worsen the accuracy of both LCL prediction models as shown in Fig. 5

All correlations were statistically significant and are shown in Fig. 4. In particular, the correlations between:

- HSI-based StO_2_ and the CLE-based FCD-A index gave a Pearson’s *R* of 0.67 (*p* value: 0.006);
- HSI-based StO_2_ and LCL gave a Spearman’s *R* of − 0.54 (*p* value: 0.04);
- The CLE-based FCD-A index and LCL gave a Spearman’s *R* of − 0.74 (*p* value: 0.001).

### LCL prediction based on HSI StO_2_

The mean error of the lactate prediction model was 2.96 ± 2 mmol/L. The median error was 2 mmol/L. A total of 95% of errors occurred for lactate values < 6.8 mmol/L.

### LCL prediction based on CLE FCD-A index

The lactate prediction model showed a mean error of 1.7 ± 1.1 mmol/L, with a median error of 1.43 mmol/L. 95% of errors occurred for lactate values < 4 mmol/L.

### Comparison between HSI StO_2_ and CLE FCD-A index LCL prediction models

**Fig. 5:**
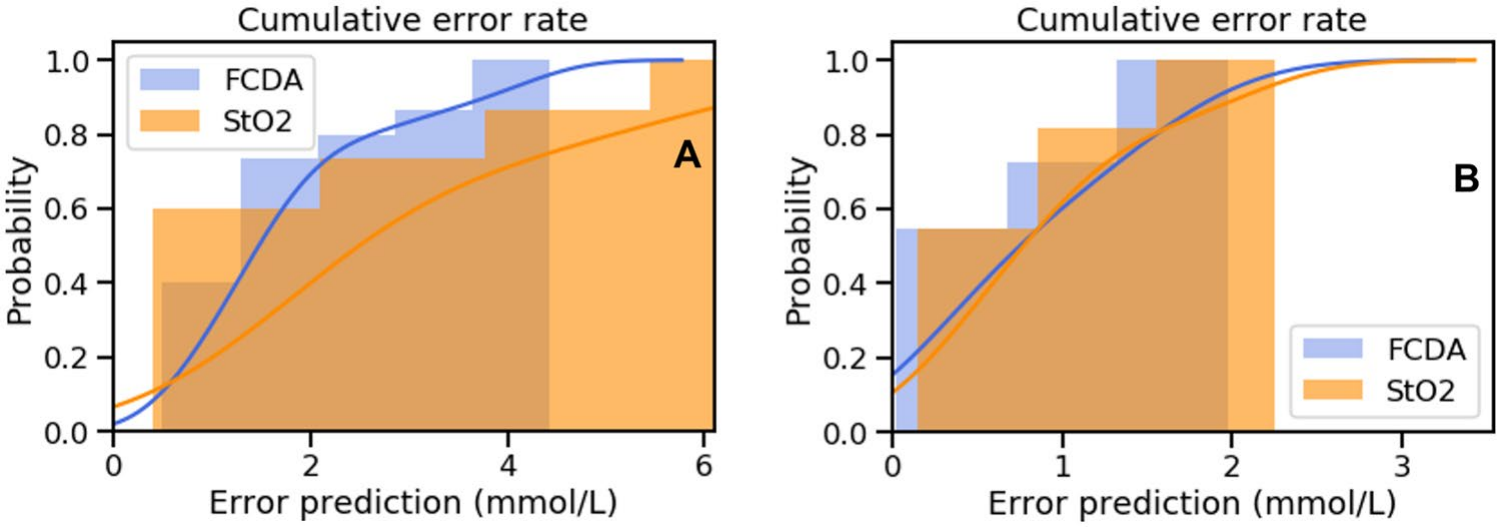
The accuracy of LCL prediction models is shown in (**A**), when considering all ROIs, and here the FCD-A index-based model is significantly more accurate than the StO_2_-based model. However, when excluding the outlying ROIs (LCL > 6 mmol/L; shown in **B**), the accuracy of both prediction models is increased and they present no statistically relevant differences

The Wilcoxon test between both prediction models resulted in the FCD-A index, LCL prediction model being significantly more accurate than the StO_2_-based one (*p* value: 0.03) (Fig. 5A).

However, as observed in previous studies using HSI to assess bowel ischemia, the accuracy of LCL prediction models increased greatly by excluding the frankly ischemic ROIs (LCL > 6 mmol/L) [12, 19]. In fact, in the exponential regression relationship between LCL and both StO_2_ and the FCD-A index, the 4 ROIs with LCL > 6 mmol/L appear as outliers (Fig. 4B, C). Consequently, the new StO_2_-based LCL prediction (excluding ROIs with LCL > 6 mmol/L) improved with a mean error of 0.88 ± 0.69 mmol/L, a median error of 0.79 mmol/L, with 95% of errors occurring for lactate values < 2 mmol/L. In addition, the new FCD-A index-based LCL prediction (excluding ROIs with LCL > 6 mmol/L) improved with a mean error of 0.82 ± 0.67 mmol/L and a median error of 0.47 mmol/L, with 95% of errors occurring for lactate values < 1.83 mmol/L.

The Wilcoxon test between those new prediction models did not show any statistically significant difference (Fig. 5B).

## Discussion

In the current study, it was possible to use HYPER in order to precisely assess gastric conduit perfusion immediately after its creation. The enhanced reality tool allowed for an accurate spatial localization of the StO_2_ information, and this subsequently allowed to scan the gastric mucosa with CLE and sample the LCL in exact correspondence of each ROI. Additionally, we observed a lower tissue perfusion, with both optical imaging techniques, at the gastric fundus (ROI-F), when compared to the gastric body (ROI-C) and to the pylorus (ROI-P). The relative ischemia present at the fundic region was also confirmed by the significant increase in LCL values within this ROI, in comparison with the remaining ROIs. These findings demonstrate that the future anastomotic region on the gastric conduit suffers from a relative ischemia and this is consistent with previous animal [24] and clinical [25, 26] studies in which perfusion was assessed during different phases of esophageal resection. Although we did not assess baseline gastric perfusion routinely in our study, we can assume that a perfusion drop from baseline levels occurred after gastric tube completion, as observed by others [24, 25]. Accordingly, in the example shown in Fig. 1, StO_2_ dropped consistently at ROI-F after slender tube creation of the stomach, whereas it changed only marginally in the other ROIs.

The marginal perfusion of esophagogastric anastomoses, resulting from a sudden disruption of the majority of the gastric fundus blood supply, is an open critical issue in digestive surgery. Consequently, in the past, several authors have used different tools to assess gastric blood flow intraoperatively, in an attempt to identify poorly perfused areas and possibly prevent anastomotic complications. Budlau et al. used a special endoscopic probe which could simultaneously act as a Doppler flow meter and spectrometer, hence allowing the concurrent quantification of mucosal blood flow and oxygen saturation [27]. In a feasibility study, the authors tested the usability of this probe in healthy individuals undergoing gastroscopy for several reasons. In a second study, the same authors successfully quantified mucosal oxygen saturation intraoperatively during esophagectomy by means of a similar spectrometric probe [26]. Recently, Irino et al. used a pulse oximetry device attached to an ear probe to assess the serosal oxygen saturation of the stomach during different steps of esophagectomy [25]. However, all of those probe-based optical techniques provide a pin-point perfusion quantification with none or minimal spatial information, and this greatly limits their routine intraoperative use. In this view, technologies with a better spatial resolution, such as laser speckle [28], fluorescence angiography (FA) [6], or thermography [29] have been explored. However, in spite of interesting results, the laser speckle’s perfusion measurement is highly variable in relation to distance and angulation from the target, and the accuracy of this technology has not yet been validated in a controlled experimental setting, using reliable ischemia biomarkers. On the other hand, FA is an easy-to-use technology, which uses an exogenous fluorophore and special near-infrared (NIR) cameras, which are becoming increasingly available in the OR, both as open and laparoscopic cameras. This technique is effective in assessing GC blood flow [6, 8, 30]. However, the evaluation is mostly based on a qualitative assessment, limiting FA reproducibility. Several FA quantification methods have been proposed [31, 32], showing promising results. However, those techniques have not been validated using robust biological ischemia markers, and as a result, their actual correlation with tissue perfusion remains unclear. Recently, a previously experimentally validated FA quantification method has been used to measure GC perfusion [33]. This quantitative FA is based on a very similar algorithm to the one previously developed and validated by our group [16, 21, 22], which computes the speed required by the injected fluorophore to reach its maximal intensity peak pixel-by-pixel. The results of this method are promising. However, this metric is not yet universally accepted and larger trials are necessary to understand its real clinical usefulness. Nishikawa et al. [29] have shown interesting results using thermal imaging during esophagectomies. In particular, the authors proposed a score based on the temperature and on the length of the gastric conduit and its supplying vessels. In their large series, they observed that patients with a lower score were more likely to develop anastomotic leakage. However, in our opinion, such a score, despite remarkable results, might be too complex to routinely reproduce outside of a research protocol.

Previously, our group used hyperspectral imaging to assess StO_2_ intraoperatively during esophagectomy [11]. In a pilot study, the feasibility of the technique was assessed, without using HYPER and subsequently only having a limited intraoperative colocalization of the perfusion information. In the current experimental study, we used HYPER, in order to localize each ROI intraoperatively, with a high degree of accuracy, and subsequently to perform a mucosal scan with CLE and LCL sampling on similar exact spots. HYPER allowed us to compare both optical techniques with one another and with local capillary lactate levels. Interestingly, our findings highlighted a good correlation between mucosal and serosal perfusion assessed using HSI and CLE, respectively. The metrics obtained by both imaging techniques correlated negatively with local capillary lactate values, confirming the biological validity of both methods. CLE correlated strongly and better than HSI to LCL levels. This finding was also confirmed by LCL prediction models, in which the FCD-A index-based prediction was significantly better than the StO_2_-based model. On the one hand, this could be explained by a better accuracy of CLE than HSI in determining blood perfusion. On the other hand, it has been demonstrated that ischemia is more extended at the mucosal than at the serosal side [14], and it is also possible that, during our experiment, mucosal ischemia was more severe and subsequently correlated better to LCL values than to the serosal value. However, this is merely a hypothesis, which cannot be demonstrated by our experimental setup. Interestingly, as previously observed [12, 19], by excluding the clearly ischemic ROIs (the ones presenting LCL values > 6 mmol/L), the precision of both LCL prediction models greatly increased. The computed prediction models excluding the highly ischemic ROIs showed no statistical difference between them. The poor accuracy of HSI to detect very ischemic regions does not affect its clinical utility, since those areas are generally well-recognized visually, and the regions difficult to assess are the ones marginally perfused, which were recognized by means of HSI with good precision.

CLE is not meant to assess bowel ischemia, since it has been conceived to perform a high-resolution in vivo histopathological assessment. However, its efficacy in identifying porcine bowel ischemia, based on micro-imaging morphological changes [16] or on the FCD-A index [17], had been previously demonstrated. In our study, CLE also proved to be a very precise tool to assess gastrointestinal perfusion. However, despite these promising results, the microscopic field of view greatly limits the usability of this device as an intraoperative perfusion assessment tool, as for all probe-based devices.

HSI is a relatively easy-to-use technique, which requires an additional camera (hyperspectral imager) providing the blood flow information as an immediate output. As previously described, this technology does not impair the surgical workflow during esophagectomy [11], since it is contactless, non-invasive and the acquisition time is less than 10 s. Differently, the intraoperative use of CLE in order to assess the mucosal perfusion of the gastric graft could be more troublesome. Firstly, as mentioned above, this technology has a microscopic spatial resolution, providing pin-point information. Secondly, it does not provide immediate perfusion results, requiring a videos post-processing. Thirdly, its utilization in the OR would either imply the intraoperative use of flexible endoscopy, with the potential hazard of manipulation of a recent esophago-gastrostomy or would require performing a gastrotomy on the GC, as in our experimental setup. Both CLE endoluminal routes are time consuming and would theoretically affect the surgical workflow; therefore, we believe that despite its greater precision and its experimental interest, CLE might not be suitable for routine intraoperative use.

The main drawbacks of our study lie in the very limited sample size, in the animal model and in the acute nature of the experiments. However, our study has the merit to demonstrate for the first time the positive strong correlation existing between hyperspectral imaging and a cutting-edge precise microscopic optical imaging technology such as CLE. This confirms the potential of HSI in identifying marginally perfused areas of gastric conduit tract and the usefulness of HYPER as a potential surgical navigation tool for this type of surgery. Despite being promising, our findings must be confirmed in the clinical setting with lager numbers in order to be able to draw any clinical and cost/benefit conclusion.
